# The role of dermatological manifestations in the early diagnosis of Fabry disease: a scoping review

**DOI:** 10.3389/fmed.2026.1899166

**Published:** 2026-10-06

**Authors:** Alessandra Maio, Jo Linda Maria Sinagra, Giulia Pascolini, Gerardo Altamura, Giovanni Di Zenzo, Annarita Panebianco

**Affiliations:** 1 University Department of Life Science and Public Health, Section of Hygiene, Università Cattolica del Sacro Cuore, Rome, Italy; 2 CEDRI, Istituto Dermopatico dell'Immacolata IDI-IRCCS, Rome, Italy; 3 Genetic Counseling Unit, Istituto Dermopatico dell'Immacolata IDI-IRCCS, Rome, Italy; 4 Rare Skin Diseases Center, Istituto Dermopatico dell'Immacolata (IDI)-IRCCS, Rome, Italy; 5 Hospital Management, Istituto Dermopatico dell’Immacolata, IDI-IRCCS, Rome, Italy; 6 Molecular and Cell Biology Laboratory, Istituto Dermopatico dell'Immacolata, IDI-IRCCS, Rome, Italy

**Keywords:** angiokeratoma, dermatological manifestations, early diagnosis, Fabry disease, hypohidrosis

## Abstract

Fabry disease (FD) is a rare X-linked lysosomal storage disorder characterized by the progressive accumulation of globotriaosylceramide in multiple organs. Despite the availability of disease-specific therapies, diagnosis is often delayed due to heterogeneous and nonspecific manifestations. This review aimed to synthesize current evidence on dermatological manifestations of FD and their potential contribution to earlier disease recognition. A scoping review was conducted following the Arksey and O'Malley framework and the PRISMA-ScR guidelines. PubMed, Scopus, and Web of Science were searched for relevant studies, and additional records were identified through snowballing of reference lists from retrieved reviews. Thirty-two records were included, comprising data on 2,209 patients, with detailed patient-level information available for 146 individuals. Dermatologists were involved in the diagnostic process in 66% of the included studies, and 69% of these reported a potential contribution of dermatological evaluation to earlier disease recognition. Angiokeratomas (88% of included studies) and hypohidrosis (50% of included studies) were the most frequently reported cutaneous manifestations. Skin biopsy or histopathological examination was reported in 59% of included studies and was the most frequently reported dermatological investigative technique. Among patients with dermatological manifestations and available diagnostic delay information, 66% had a reported diagnostic delay of 1–10 years. These findings suggest that dermatological manifestations may contribute to earlier recognition of FD. However, they should be interpreted cautiously given the descriptive nature of the available evidence, the absence of an appropriate comparator group, and the potential influence of selection and publication bias.

## 1 Introduction

Rare diseases, while individually infrequent, collectively impact a significant proportion of the global population with over 7,000 identified conditions affecting an estimated 25–30 million adults in the United States alone (1). Many of these conditions have a genetic basis, and their investigation has led to significant insights into human pathophysiology, paving the way for innovative treatments and precision medicine approaches (2).

Fabry disease (FD) exemplifies the diagnostic challenges posed by multisystemic rare disorders. This X-linked lysosomal storage disorder results from variants in the *GLA* gene, which lead to deficient *α*-galactosidase A activity and subsequent accumulation of globotriaosylceramide (Gb3) in multiple tissues (3). The progressive nature of FD affects the kidneys, cardiovascular system, nervous system, and skin, often culminating in severe morbidity if untreated. Despite the availability of disease-specific treatments, including enzyme replacement therapy (ERT) and pharmacological chaperone therapy, delays in diagnosis persist, hindering optimal clinical management (4).

FD frequently manifests during childhood, although the diagnosis is often delayed until adulthood because its early signs are nonspecific and underrecognized. Among the earliest clinical manifestations, hypohidrosis, hyperhidrosis or anhidrosis may develop in early childhood as a consequence of autonomic dysfunction, leading to impaired thermoregulation and heat intolerance (4). Angiokeratomas generally appear later, most commonly during late childhood or adolescence, and progressively increase in number and distribution with advancing age, particularly in patients with the classic phenotype. Although angiokeratomas are a characteristic dermatological feature of FD, they are not pathognomonic and may also occur in several other disorders, including fucosidosis, sialidosis, Kanzaki disease (*α*-N-acetylgalactosaminidase deficiency), *β*-mannosidosis, and other lysosomal storage disorders (5). These manifestations often coexist with neuropathic pain, gastrointestinal symptoms, and corneal verticillata, providing important clinical clues that should raise suspicion for FD (4). Recognition of these pediatric features is of particular importance, as increasing evidence indicates that pathological glycolipid accumulation and early organ involvement begin well before the onset of irreversible cardiac, renal, or cerebrovascular complications.

In addition to clinical suspicion based on characteristic manifestations, several complementary screening approaches are now employed. Newborn screening programs, implemented in several countries and regions, have demonstrated that FD is more prevalent than previously estimated and facilitate presymptomatic diagnosis (4). Screening of high-risk populations, including individuals with unexplained left ventricular hypertrophy, chronic kidney disease, cryptogenic stroke, or small-fiber neuropathy, has proven to be an effective strategy for detecting previously unrecognized cases. Once an index patient is identified, family cascade testing remains one of the most efficient and cost-effective diagnostic approaches, enabling the identification of affected relatives across multiple generations through targeted genetic testing and appropriate clinical evaluation. Biochemical assessment has also advanced with the widespread use of globotriaosylsphingosine (lyso-Gb3), a sensitive biomarker that complements measurement of *α*-galactosidase A enzyme activity and *GLA* gene sequencing (4). Together, these complementary approaches have improved diagnostic yield and may facilitate earlier recognition of FD across diverse clinical settings.

However, several factors contribute to the delayed recognition of FD. Its broad spectrum of clinical manifestations and variable phenotypic expression complicate early identification. Many initial symptoms, such as neuropathic pain, proteinuria, and cardiovascular abnormalities, are nonspecific and frequently misinterpreted as indications of more common conditions, resulting in misdiagnosis and inappropriate treatment (5). Additionally, limited awareness among healthcare providers further exacerbates diagnostic delays (6). On the other hand, as an X-linked disorder, heterozygous females can be symptomatic when the inactivation preferentially affects the wild type X-chromosome in the majority of their cells, in a process called “skewing” of X inactivation. Thus, the heterozygous female population presents an additional challenge, as residual enzyme activity may lead to an attenuated phenotype, often necessitating comprehensive genetic testing for definitive diagnosis (7).

Angiokeratomas, along with hypohidrosis or anhidrosis, are the most prevalent cutaneous manifestations and may serve as early indicators of systemic involvement. Although the association between dermatological manifestations and FD is well established and numerous cases have been reported in the literature in which the diagnosis was established by dermatologists and prompted by the cutaneous manifestations of the disease, the potential contribution of dermatological assessment to earlier disease recognition remains incompletely characterized. Our experience at our tertiary dermatology referral center supports this view, as the number of FD diagnoses remains disproportionately low compared with the estimated prevalence of the disease. This suggests that dermatology-based case identification is still underutilized and that greater awareness among dermatologists may facilitate earlier recognition of FD.

This scoping review aims to consolidate current evidence on the dermatological manifestations of FD and their potential contribution to earlier disease recognition and their relationship with diagnostic timing. Furthermore, it will explore the potential implementation of dermatology-led referral pathways as a means to support earlier recognition and multidisciplinary management of affected individuals.

## 2 Material and methods

### 2.1 Study design

We conducted a scoping review following the Arksey and O'Malley framework (8), ensuring adherence to the PRISMA-ScR (Preferred Reporting Items for Systematic Reviews and Meta-Analyses extension for Scoping Reviews) checklist (Supplementary Table S1).

Our research question was structured using the PCC framework:

- Population (P): Patients with FD, with emphasis on those with dermatologic manifestations.
- Concept (C): Dermatologic symptoms of FD (angiokeratomas, hypohidrosis/anhidrosis, lymphedema, other skin changes) and their role in early diagnosis and diagnostic timing.
- Context (C): Clinical diagnosis in dermatology, diagnostic pathways for rare diseases, and potential implementation of targeted dermatology-based screening.

The primary research question addressed in this review was: “What are the dermatological manifestations of FD reported in the literature, and what is their potential contribution to early diagnosis and diagnostic timing?”.

A detailed version of the study protocol is publicly available on the Open Science Framework (DOI: 10.17605/OSF.IO/CHJD5).

### 2.2 Search strategy

We conducted a comprehensive search of three major databases—PubMed, Scopus, and Web of Science—using the following search term: (“Fabry Disease” OR “Anderson-Fabry disease” OR “alpha-galactosidase A deficiency”) AND (“Skin Manifestations” OR “Angiokeratoma” OR “Hypohidrosis” OR “Anhidrosis” OR “Dermatological symptoms” OR “Skin lesions” OR “Lymphedema” OR “Acroparesthesias”) AND (“Early Diagnosis” OR “Diagnosis, Differential” OR “Time to Diagnosis” OR “Diagnostic delay” OR “Dermatology pathway”).

The search strategy was adapted to the characteristics of each database and combined Medical Subject Headings (MeSH), where available, together with free-text keywords**.**

In addition, we performed a snowballing procedure, screening the reference lists of the reviews identified through the database search. This step aimed to capture potentially relevant studies that might not have been retrieved electronically due to indexing or terminology differences.

### 2.3 Eligibility criteria

Studies were eligible if they involved patients with confirmed FD, assessed dermatological manifestations and their potential contribution to earlier disease recognition or diagnostic timing, and provided relevant clinical data. Observational studies, case reports, case series, and reviews addressing the potential diagnostic contribution of dermatological signs were included. Only studies published in English were considered. Studies without confirmed FD diagnosis, lacking dermatological data, focusing exclusively on non-dermatological aspects, or without accessible full text were excluded, together with editorials, opinion papers, and abstracts without full-text availability.

### 2.4 Screening process

All records retrieved from both the database search and the snowballing procedure were imported into a reference manager (https://www.rayyan.ai), where duplicates were removed. The screening process was carried out in two consecutive stages. First, titles and abstracts were examined to assess whether each record met the predefined eligibility criteria. Subsequently, the full texts of potentially relevant articles were retrieved and reviewed in detail to confirm eligibility. Any conflicts regarding inclusion were resolved through discussion until consensus was reached.

### 2.5 Data charting

Data from all included records were charted at three complementary levels, using a standardized form aligned with the final tables.

At the record-level synopsis, we captured: role of dermatology in the initial diagnosis; impact on early diagnosis; consideration of dermatology-led screening; and a concise narrative of the main findings and conclusions.

At the study level, we extracted: study type; sample size; mean or median age when reported; typical dermatological manifestations with prevalence where available; other symptoms/signs with prevalence where available; and skin imaging and histopathological findings.

At the patient level for case reports and case series, we extracted: sex; typical dermatological manifestations; age at onset of dermatological manifestations; other systemic symptoms/signs; age at onset of other systemic symptoms/signs; dermatological diagnostic methods (clinical inspection, dermoscopy, biopsy/histology, electron microscopy, and other skin imaging where applicable); and diagnostic delay.

When prevalence was not explicitly reported but numerators and denominators were available, percentages were calculated; when data were unavailable, items were marked as “not applicable” (NA). Diagnostic delay was recorded as reported; when both age at onset and age at diagnosis were available, it was computed as their difference.

### 2.6 Statistical analysis

Statistical analysis was limited to descriptive summaries, with categorical variables presented as absolute frequencies and percentages. No inferential statistical analyses were performed, and subgroup distributions were presented for descriptive purposes only. All analyses were performed using Stata version 18.

## 3 Results

### 3.1 Study algorithm

A total of 386 records were identified through database searches (Figure 1). After removal of duplicates and screening by title and abstract, 47 reports were assessed for full-text eligibility. Of these, 27 records were included in the evidence synthesis, while 6 review articles were retained exclusively for citation mining and were not included in the evidence synthesis. Screening the reference lists of these reviews identified 5 additional eligible records. Overall, 32 records were included in the scoping review evidence synthesis: 27 identified through database searches and 5 through review snowballing.

**Figure 1:**
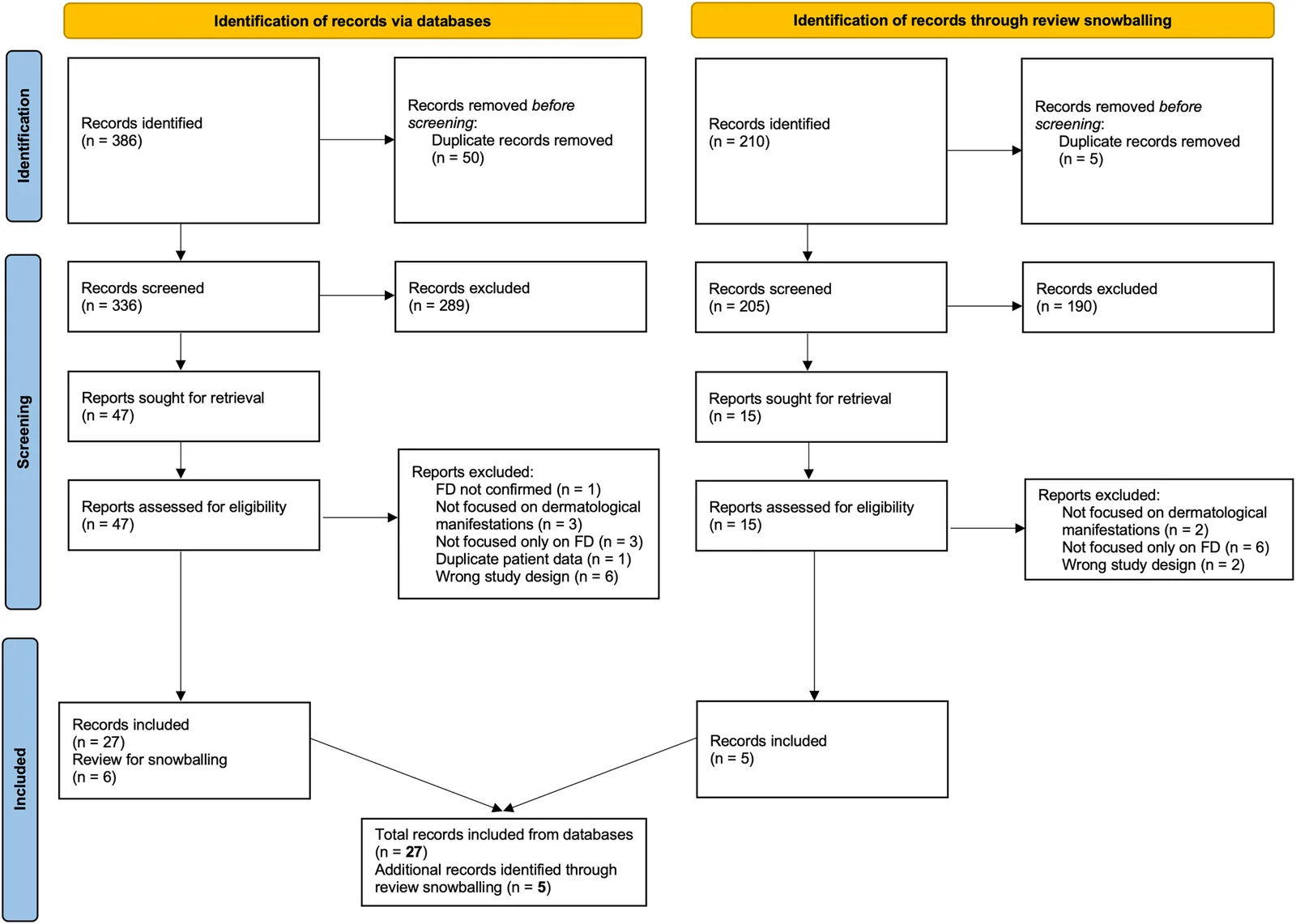
Flowchart of included records.

### 3.2 Study-level analysis

Among the 32 studies included, the role of dermatology in the diagnostic process of FD varied across reports (Table 1). Dermatology was involved in 66% of the included studies, had a partial role in 19%, and was not involved in 13% of cases; in 3%, the role was not clearly specified. Regarding early diagnosis, 69% of the included studies reported that dermatological assessment contributed to earlier disease recognition, whereas 28% did not report such a contribution, and 3% provided unclear information. Consideration of dermatology-led screening was reported in 78% of studies, while 22% did not provide clear or sufficient information on this aspect.

**Table 1:** Dermatological manifestations and their impact on early diagnosis of FD.

References	Authors, Year	Role of dermatology in initial diagnosis	Contribution to early diagnosis	Consideration of dermatology-led screening	Main findings & conclusions
(9)	Ahlmén et al., 1982	No—skin signs noted but not decisive	No—diagnosis via renal/metabolic pathway	Unclear	Skin signs under-recognized; diagnosis based on renal/biopsy findings; early recognition important but dermatology not leveraged for FD.
(10)	Anker et al., 2023	Yes—derm exam + dermoscopy key	Yes—improved early detection (atypical lesions may delay)	Yes	Angiokeratomas common but heterogeneous; dermoscopy/advanced imaging (MSI, NLM) enhance early recognition of FD.
(11)	Choudhury et al., 2005	Yes—pediatric derm referral; biopsy	Yes—dx at 11 y before organ involvement	Yes	Atypical petechiae-like angiokeratomas require biopsy/work-up for FD to reduce delay.
(12)	Corry et al., 2011	Yes—solitary angiokeratoma triggered work-up	Yes—enabled index dx and family screening	Yes	Even a single angiokeratoma can be the only clue; vigilance in derm clinics recommended for FD.
(13)	Da Silva et al., 2014	Yes—angiokeratomas among first signs	Yes—early signs could have shortened delay	Yes	Dermatologists should recognize angiokeratomas for timely referral and FD diagnosis in relatives.
(14)	de Groot, 1970	Yes—pediatric/adult angiokeratomas key	Yes—childhood recognition enables early dx	Yes	Early pediatric angiokeratomas are critical clues; awareness improves timely FD diagnosis.
(15)	de Jesus et al., 2018	Yes—dermatologist recognized genital angiokeratoma	Yes—clue to dx; ERT started	Yes	Genital angiokeratomas in women can be pivotal; dermatology central to early FD detection.
(16)	Dean & Starr, 2014	No—diagnosis after renal biopsy	No—misdiagnosis delayed recognition	Unclear	Lymphedema may be atypical cutaneous manifestation; consider FD in unexplained/familial lymphedema.
(17)	Dilorenzo et al., 1969	Yes—recognition/biopsy of lesions	Yes—cutaneous clue enabled dx	Yes	Angiokeratomas are key; derm exam + biopsy crucial for early FD recognition.
(18)	Franques et al., 2017	No—recognition mainly neurological/systemic	No—cutaneous signs not sufficient alone	Yes	Dyshidrosis/angiokeratomas are early indicators that should prompt FD evaluation.
(19)	Furujo et al., 2013	Yes—angiokeratomas raised suspicion	Yes—allowed timely ERT	Yes	Pediatric angiokeratomas/hypohidrosis are important early signs; ERT improved symptoms in FD.
(20)	Galanos et al., 2002	Yes—in some probands	No—long delays despite early signs	Yes	Angiokeratomas/anhidrosis prevalent; awareness in children emphasized to avoid ≥10 y delays in FD.
(21)	Hsu et al., 2019	Yes—hypohidrosis/angiokeratomas key	Yes—earlier recognition could prevent complications	Yes	Systematic derm exam for hypohidrosis/vascular lesions recommended to detect FD early.
(22)	Jung et al., 2015	Yes—hypohidrosis + angiokeratoma triggered suspicion	Yes—dx before organ involvement; early ERT	Yes	Sweating abnormalities may be first manifestation; prompt recognition enables treatment in FD.
(23)	Kotnik et al., 2005	Yes—derm exam/biopsy confirmed	Yes—childhood lesions could have enabled earlier dx	Yes	Shows diagnostic value of dermatological signs; early recognition supports timely ERT in FD.
(24)	Larralde et al., 2004	Yes—derm exam of angiokeratomas	Yes—potential to shorten ∼10.8 y delay (males)	Yes	Angiokeratomas/hypohidrosis are key early signs; carriers may show isolated lesions; FD awareness essential.
(25)	Li et al., 2023	Yes—pediatric cutaneous signs raised suspicion	Yes—pediatric screening advocated	Yes	Early pediatric angiokeratomas/hypohidrosis; earlier derm recognition could avoid complications; start ERT sooner in FD.
(26)	Lidove et al., 2006	Partial—sweating abnormalities noted by dermatologist in some	Yes—early, esp. in females/children	Yes	Hyperhidrosis can be an early FD sign; consider FD in unexplained hyperhidrosis.
(27)	Lidove et al., 2012	Partial—direct diagnosis via derm in 25.9%	No—substantial delays persisted	Unclear	Cutaneous signs contribute but are not specific; broad awareness needed to reduce FD delays.
(28)	Lorenz et al., 2003	No—diagnoses mainly nephrology-led	No—often after renal involvement	Unclear	Cutaneous signs frequent but not leveraged; underdiagnosis and specialty-based detection discussed for FD.
(29)	Marchesoni et al., 2010	Partial—some index cases via derm, many missed	No—marked delays (≈15 y males; 25 y females)	Unclear	Early features incl. derm signs often missed; greater awareness could reduce FD misdiagnosis.
(30)	Martin et al., 2007	Partial—dermatologist consulted for vascular signs	No—atypical presentation; tests decisive	Unclear	Heterozygous woman with ischemia/acroparaesthesia; high suspicion needed even without classic lesions of FD.
(31)	Mehta et al., 2004	Partial—dermatology among diagnosing specialties	No—mean delays 13–16 y	Yes	Angiokeratomas frequent/early; broader derm awareness could reduce diagnostic delay in FD.
(32)	Menkes, 1999	Yes—classic bathing-trunk lesions recognized	Yes—rapid dx with enzyme/EM	Yes	Recognizing characteristic lesions avoids unnecessary testing; enables counseling in FD.
(33)	Mungan et al., 2015	Yes—first presentation to dermatology; biopsy	Yes—early dx before organ damage	Yes	Derm findings can suffice to trigger work-up; ERT improved symptoms; lesions/sweating less responsive in FD.
(34)	Nagai-Sangawa et al., 2022	Yes—derm-led hypohidrosis clinic identified cases	Yes—early dx; family screening positive	Yes	Screening among unexplained hypohidrosis patients efficiently detects FD.
(35)	Nelis & Jacobs, 1989	Yes—derm recognition of angiokeratomas	Yes—dx after years of GI symptoms thanks to derm	Yes	Cutaneous signs decisive even with atypical GI onset; supports derm-led recognition of FD.
(36)	Orteu et al., 2007	Yes—first diagnosis by dermatologist in 14% M/2% F	Yes—earlier recognition when derm involved	Yes	Cutaneous signs correlate with severity; dermatologists key yet underutilized for FD.
(37)	Ries et al., 2003	Partial—angiokeratomas key when present	Yes—when present, allow prompt dx	Yes	Pediatric phenotype (pain, hypohidrosis, angiokeratomas, GI); recommend systematic skin/eye exams for FD.
(38)	Siedler et al., 2019	Unclear	Unclear	Unclear	Dyshidrosis linked to reduced PREP amplitudes in women; supports diagnostic potential of small-fiber/autonomic testing in FD.
(39)	Tokuriki et al., 2013	Yes—derm biopsy/EM of cherry angiomas	Yes—early dx before organ involvement	Yes	Cherry angiomas can be a presentation; EM assists early confirmation; ERT initiated in FD.
(40)	Werninghaus et al., 1995	Yes—derm exam + biopsy with EM confirmed	Yes—derm signs led to recognition	Yes	Unusual telangiectatic angiectases (not classic angiokeratomas) diagnosed via skin biopsy/EM; FD is a “great imitator”.

Derm, dermatological; FD, Fabry disease; ERT, enzyme replacement therapy; EM, electron microscopy; GI, gastrointestinal; FUO, fever of unknown origin; MSI, multispectral imaging; NLM, nonlinear microscopy; PREP, pain-related evoked potentials.

● **Role of dermatology in initial diagnosis:**

Yes = dermatology was directly responsible for the initial suspicion/confirmation of FD.

No = dermatology had no role in the diagnostic process.

Partial = dermatology contributed in some cases or indirectly but was not consistently decisive.

Unclear = the role of dermatology is not clearly reported or cannot be determined from the study.

● **Impact on early diagnosis:**

Yes = dermatological findings allowed diagnosis before major organ involvement or shortened diagnostic delay.

No = dermatological signs were present but did not lead to earlier recognition; diagnosis occurred late or via other specialties.

Unclear = the impact of dermatological signs on diagnostic timing is not clearly reported or cannot be determined.

● **Consideration of dermatology-led screening:**

Yes = authors explicitly recommend or strongly support systematic dermatology-based screening/referral for FD.

No = authors explicitly state dermatology is not sufficient or not relevant for screening.

Unclear = no explicit statement or recommendation is provided.

● **Main findings & conclusions:** concise synthesis of key study messages, with dermatology focus.

Study designs varied across the included records (Table 2). Specifically, 44% of the 32 included records were case reports, 28% case series, 3% case–control studies, 6% cross-sectional studies, 13% cohort studies, and 6% registry-based or mixed-design records. Overall, the included records collectively reported data on 2,209 patients, with substantial variation across study designs. Cohort studies contributed markedly larger samples than most other designs (mean: 290 patients), whereas registry-based and mixed-design records included the largest populations overall (mean: 399 patients).

**Table 2:** Summary of included records.

References	Authors, Year	Study type	N total	N male	N female	Mean age (years)	Dermatological manifestations	Systemic involvement/ Family history	Skin imaging & histopathology
Cohort studies									
(29)	Marchesoni et al., 2010	Retrospective observational	45	25	20	M 9.8 F 10.9	Angiokeratomas Hypohidrosis/anhidrosis	Neurological GI Cerebrovascular Vestibular	NA
(31)	Mehta et al., 2004	Prospective cohort	366	201	165	M 35.5 ± 13.1 F 41.4 ± 17.1	Angiokeratomas (M 78%; F 50%)	Neurological (M 76%; F 64%) GI (M 55%; F 50%) Renal (M 44%; F 33%) Cardiac (M 69%; F 65%) Cerebrovascular (M 12%; F 27%) Ocular (M 62%; F 53%) Auditory (M 57%; F 47%) Fatigue (∼25%)	NA
(36)	Orteu et al., 2007	Prospective cohort	714	345	369	M 35 ± 15 F 38 ± 18	Angiokeratomas (M 66%; F 36%) Telangiectasia (M 23%; F 9%) Hypohidrosis (M 53%; F 28%) Anhidrosis (M 25%; F 4%) Hyperhidrosis (M 6%; F 13%) Lymphedema (M 16%; F 6%) Heat intolerance (M 44%; F 30%)	NA	NA
(37)	Ries et al., 2003	Prospective cohort	35	15	20	12.6	Angiokeratomas (M53%; F 30%) Hypohidrosis (M 93%; F 25%)	Neurological (M 67%; F 65%) GI (M 40%; F 20%) Ocular (M 73%; F 70%) Cardiac (M 13%; F 20%) Renal (M13%; F 15%)	NA
Cross-sectional studies									
(10)	Anker et al., 2023	Cross-sectional	26	12	14	38 ± 20.8	Angiokeratomas (92%) Hypohidrosis/anhidrosis (42%) Hyperhidrosis (27%) Lymphedema (19%) Other: synophrys (15%)	Ocular (69%) Cardiac (65%) Renal (42%) Neurological (62%) Cardiovascular (38%) Stroke (15%) GI (42%) Pulmonary (15%) Auditory (19%) Fatigue (50%)	Dermoscopy: lacunae, dotted vessels, whitish veil Biopsy: dilated vessels, acanthosis, hyperkeratosis
(34)	Nagai-Sangawa et al., 2022	Cross-sectional + case report	17	16	1	NA	Hypohidrosis Angiokeratomas	Neurological Ocular Renal Cardiac	Biopsy: angiokeratoma with EM lamellar inclusions
Case-control studies									
(38)	Siedler et al., 2019	Case-control	58	26	32	43	Hypohidrosis/anhidrosis (M 58%; F 22%)	GI (10%) Pain (62%) Renal (14%) Cardiac	Biopsy: IENFD
Registry-based and mixed studies									
(26)	Lidove et al., 2006	Mixed: case series + prospective cohort + registry	740	NA	NA	NA	Hyperhidrosis (FOS 9.2%; F 11.9%; M 6.4%) Angiokeratoma	Neurological GI Cardiac Cerebral MRI lesions Hearing	NA
(27)	Lidove et al., 2012	Observational mixed	58	23	35	Overall 35.8 M 27.6 F 42.2	Angiokeratomas (20.7%) Raynaud's (1.7%) Hypohidrosis/anhidrosis (3.4% F)	Neurological (25.9%) Cardiac (17.2%) Cerebrovascular (10.3%) Renal (8.6%) Ocular (10.3%)	NA
Case series									
(9)	Ahlmén et al., 1982	Case series	3	3	0	NA	Hypohidrosis Papules	Neurological Renal Cardiovascular Ocular GI	Biopsy: one lesion misinterpreted as lichen ruber
(13)	Da Silva et al., 2014	Case series	3	2	1	NA	Angiokeratomas	Neurological Ocular Auditory Renal GI	Biopsy: umbilical angiokeratoma (capillary ectasia, epidermal thickening)
(14)	de GROOT, 1970	Case series	12	5	7	NA	Angiokeratomas	Neurological Ocular	Histology: lipid deposition in vessel walls (Sudan Black); angiokeratoma biopsy
(18)	Franques et al., 2017	Case series	18	15	3	47.5	Hypohidrosis (67%) Angiokeratomas (22%)	Neurological Ocular (44%) Cardiac (78%) Renal (28%) GI (11%)	NA
(19)	Furujo et al., 2013	Case series	2	2	0	NA	Angiokeratomas Hypohidrosis/anhidrosis	Ocular Neurological GI	NA
(20)	Galanos et al., 2002	Case series	67	29	38	M 26.1 F 48.3	Angiokeratomas (M 93%; F 13%) Anhidrosis (M 93%; F 11%)	Neurological (M 100%; F 53%) Renal (M 69%; F 21%) Cardiac (M 28%; F 26%) Cerebrovascular (M 31%; F 5%) Ocular (M 96%; F 76%)	Biopsy: some cases
(24)	Larralde et al., 2004	Case series	11	6	5	M 23 F 49.9	Angiokeratomas (M 83%; F 40%) Hypohidrosis (M 100%; F 0%)	Neurological GI Renal Ocular	Biopsy EM: lamellar inclusions, dilated dermal vessels, acanthosis, hyperkeratosis
(25)	Li et al., 2023	Case series	10	7	3	11.7	Angiokeratomas (71% symptomatic) Hypohidrosis/anhidrosis (71%)	Neurological GI Ocular Auditory Renal Cardiac Musculoskeletal Endocrine	NA
(28)	Lorenz et al., 2003	Case series	9	6	3	NA	Angiokeratomas (66.7%) Hypohidrosis (44.4%)	Ocular (88.9%) Neurological (66.7%) Renal (77.8%) Cardiac (66.7%) Auditory	NA
Case report									
(11)	Choudhury et al., 2005	Case report	1	1	0	NA	Angiokeratomas (atypical widespread distribution)	Neurological	Biopsy: dilated dermal vessels (angiokeratoma)
(12)	Corry et al., 2011	Case report	1	1	0	NA	Angiokeratoma (solitary)	Renal	Biopsy: angiokeratoma confirmed
(15)	de Jesus et al., 2018	Case report	1	0	1	NA	Genital angiokeratoma	Neurological Family history	Dermoscopy: blue-red lakes Biopsy: vascular dilatation with acanthosis
(16)	Dean & Starr, 2014	Case report	1	1	0	NA	Lymphedema	Neurological Renal GI Ocular Cardiovascular	NA
(17)	Dilorenzo et al., 1969	Case report	1	1	0	NA	Angiokeratomas (bathing-trunk)	Cerebrovascular Cardiac Neurological Ocular Renal	Biopsy: dilated dermal vessels; Sudan Black B + lipid
(21)	Hsu et al., 2019	Case report	1	1	0	NA	Hypohidrosis Angiokeratomas (genital) Telangiectasia	Neurological GI Autonomic Ocular Cerebrovascular	Biopsy: preserved sweat glands despite hypohidrosis
(22)	Jung et al., 2015	Case report	1	1	0	NA	Hypohidrosis Angiokeratomas	Neurological	Biopsy (palm): normal Biopsy (elbow): dilated papillary dermal capillaries
(23)	Kotnik et al., 2005	Case report	1	1	0	NA	Angiokeratomas (diffuse) Hypohidrosis	Neurological Musculoskeletal Cardiac Renal Ocular Cerebrovascular	Biopsy (abdomen): hyperkeratosis, dilated dermal vessels
(30)	Martin et al., 2007	Case report	1	0	1	NA	Other: digital ischemia with non-blanching purple discoloration	Neurological Cardiovascular GI Family history	Biopsy (acral): unremarkable EM (random skin): no lamellar bodies
(32)	Menkes, 1999	Case report	1	1	0	NA	Angiokeratoma corporis diffusum	Neurological Renal Ocular Autonomic	EM: zebra bodies
(33)	Mungan et al., 2015	Case report	1	1	0	NA	Angiokeratomas Hypohidrosis/anhidrosis	Musculoskeletal Autonomic	Biopsy: hyperkeratosis; dilated dermal vessels
(35)	Nelis & Jacobs, 1989	Case report	2	2	0	NA	Angiokeratomas	GI Neurological Ocular Dental	NA
(39)	Tokuriki et al., 2013	Case report	1	1	0	NA	Papules Cherry angiomas	Neurological Autonomic Family history	Biopsy: cherry angioma pattern EM: dense/lamellar deposits
(40)	Werninghaus et al., 1995	Case report	1	1	0	NA	Telangiectases Angiokeratomas	Renal Cardiovascular Endocrine Auditory Neurological Pulmonary	Biopsy: dermal vessels with lipid deposits EM: lamellar inclusions

FD, Fabry disease; GI, gastrointestinal; NA, not available; EM, electron microscopy; IENFD, intraepidermal nerve fiber density.

The distribution of dermatological manifestations across the 32 included records is shown in Figure 2. Angiokeratomas were the most frequently reported cutaneous feature, described in 88% of the included records. Hypohidrosis appeared in 50% of records, whereas hyperhidrosis, telangiectasia, and lymphedema were each reported in 9% of records. Other dermatological manifestations were documented in 19% of records. Overall, while angiokeratomas and altered sweating emerged as the most consistently described cutaneous signs, other manifestations were comparatively uncommon within the available literature.

**Figure 2:**
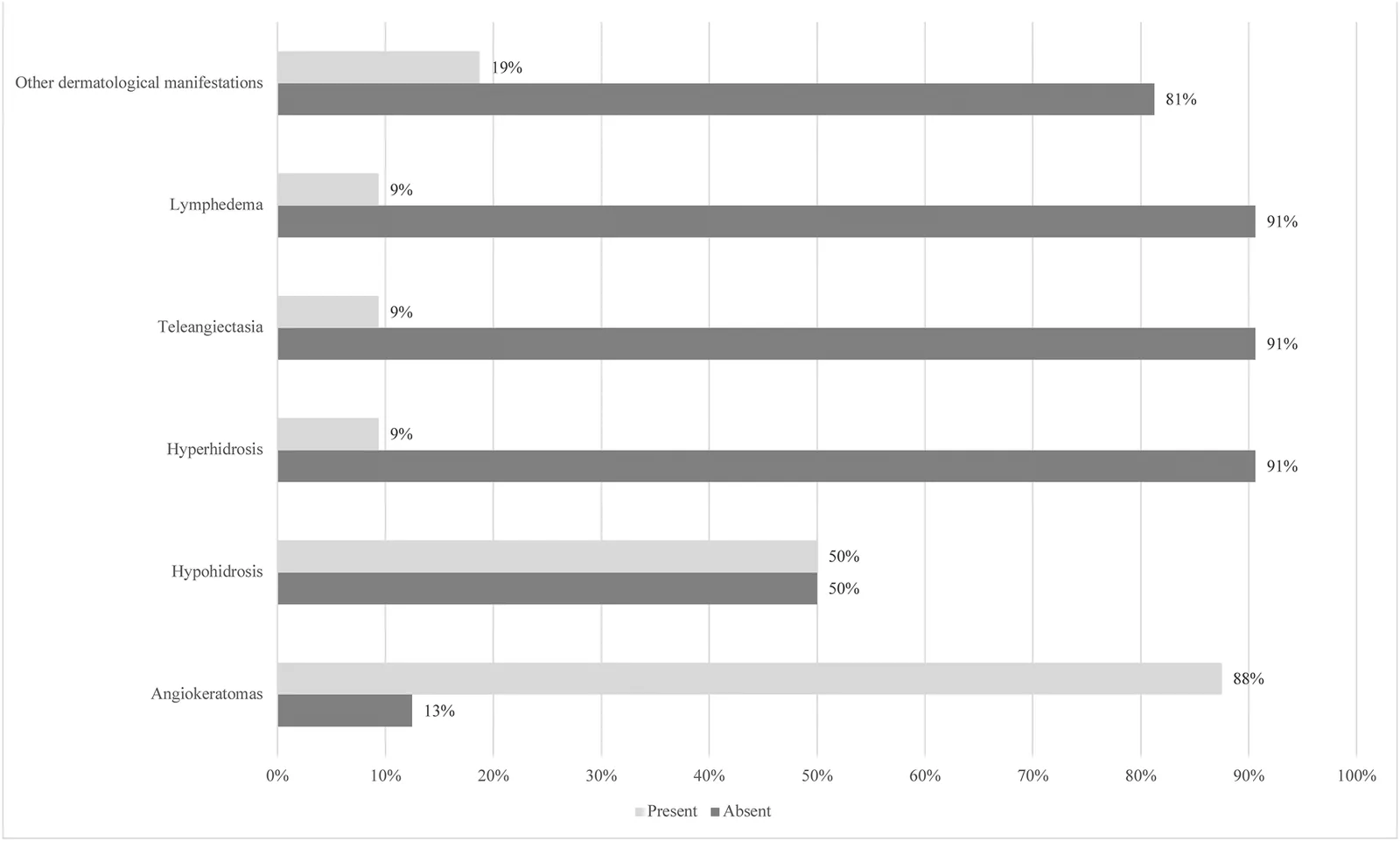
Distribution of dermatological manifestations across the included records. Percentages were calculated using the included records (*n* = 32) as the denominator. Percentages may not sum to 100% due to rounding.

Gastrointestinal involvement was the most frequently reported systemic feature, appearing in 91% of records, followed by neurological manifestations (88%) (Figure 3). Renal and ocular involvement were reported in 59% and 56% of records, respectively. Cardiac manifestations were described in 38% of records, whereas cerebrovascular complications were reported in 25%. Auditory manifestations were documented in 19% of records, and autonomic involvement in 13%.

**Figure 3:**
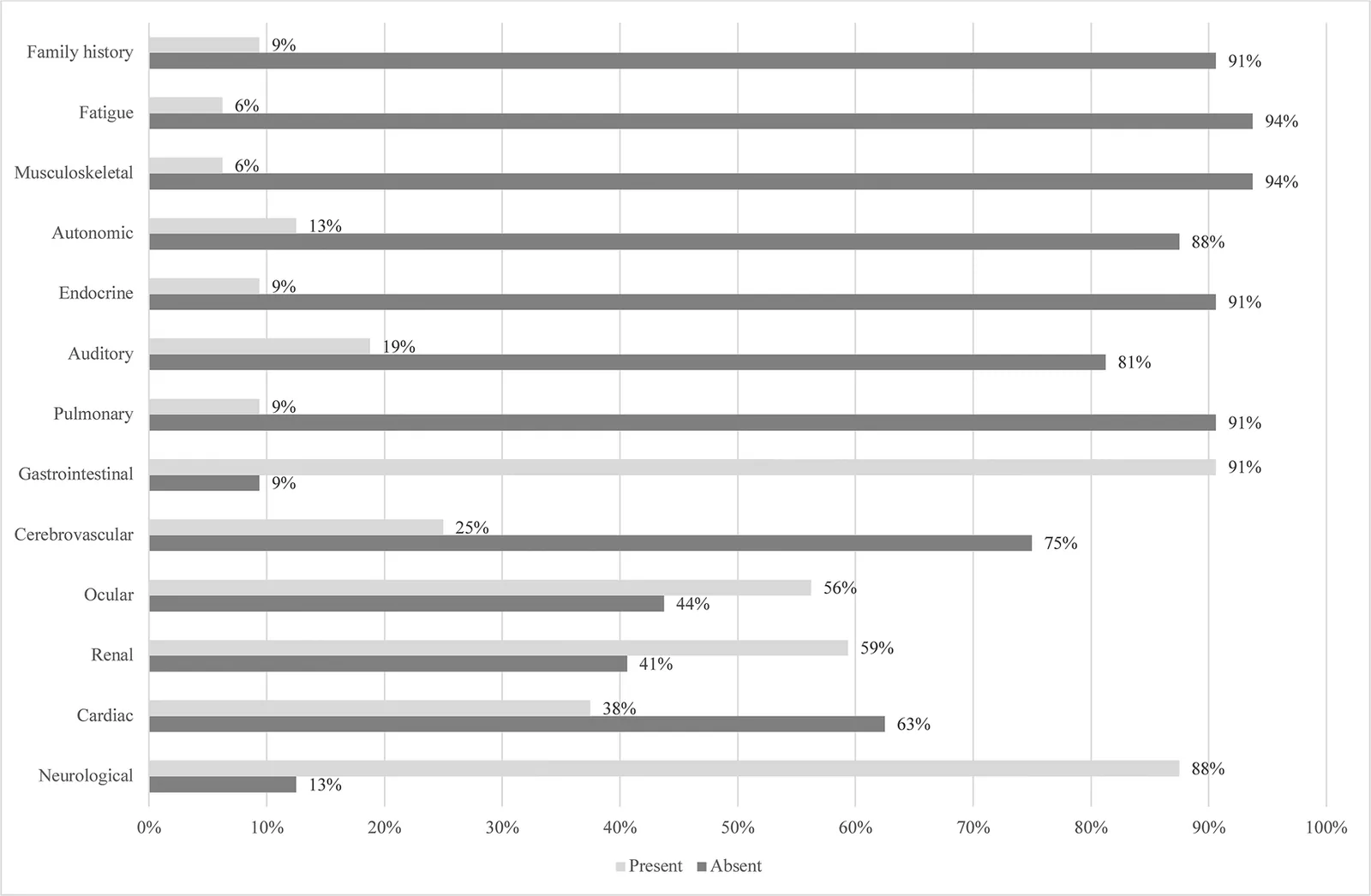
Distribution of family history, fatigue and organ-specific manifestations across the included records. Percentages were calculated using the included records (*n* = 32) as the denominator. Percentages may not sum to 100% due to rounding.

Pulmonary and endocrine manifestations were each reported in 9% of records, while musculoskeletal manifestations and fatigue were less frequently documented (6% each). Skin biopsy or histopathological examination was the most frequently reported dermatological investigative technique, being described in 59% of the included records (Figure 4). Electron microscopy was described in 19% of records, while dermoscopy was reported infrequently (6%). Other dermatologic imaging modalities were mentioned in 9% of records.

**Figure 4:**
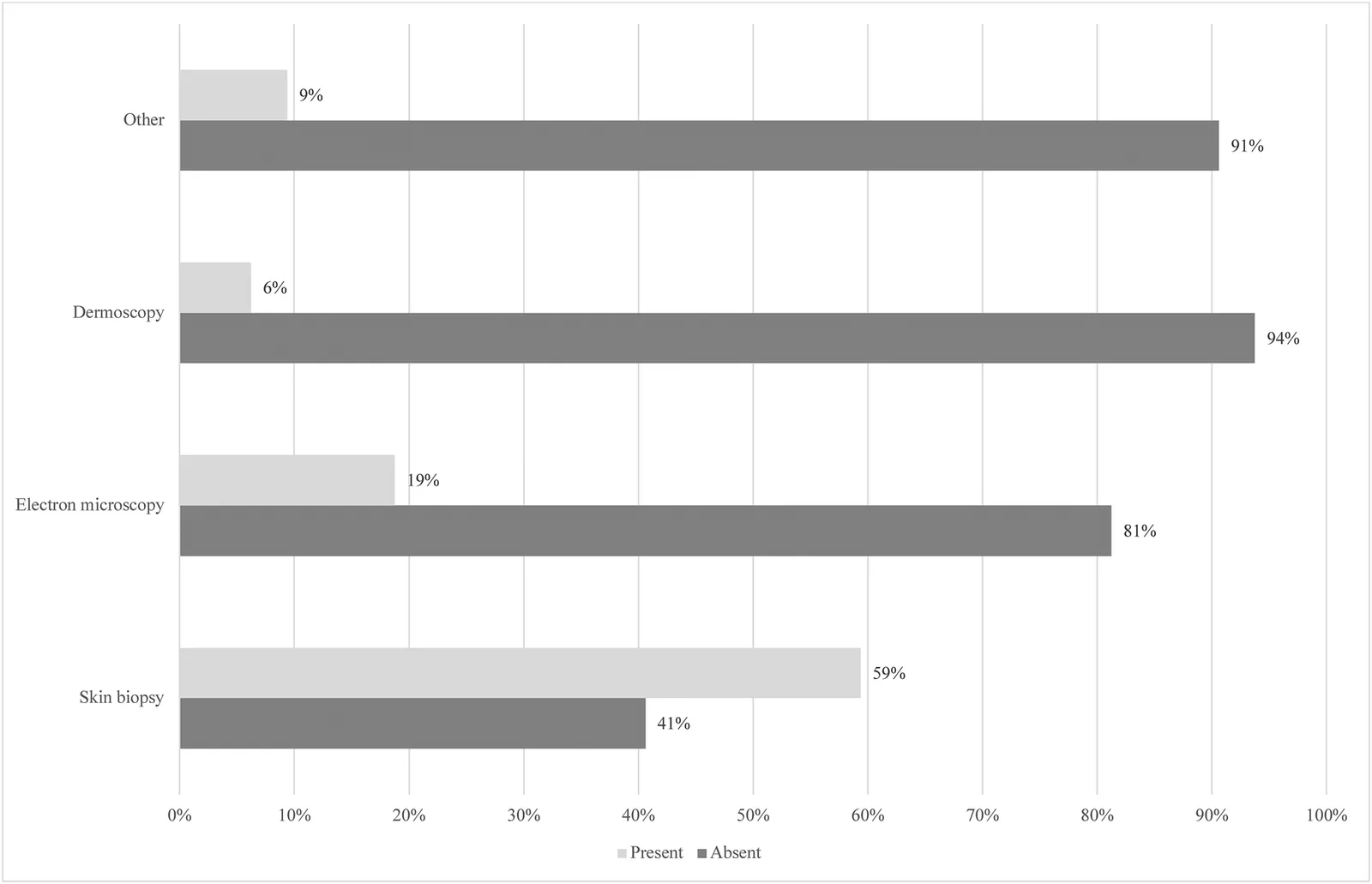
Distribution of diagnostic dermatologic methods across the included records. Percentages were calculated using the included records (*n* = 32) as the denominator.

### 3.3 Patient-level analysis

Patient-level characteristics extracted from case reports and case series are summarized in supplementary Table S2, including sex distribution, timing of dermatological and systemic symptom onset, relative sequencing of clinical manifestations, and reported diagnostic delays. Across the 146 individual patients described, 68% were male and 32% female (Table 3). Dermatological manifestations most commonly began during adolescence (25%) or late childhood (15%), although onset age was not reported in 41% of cases. Systemic manifestations, instead, more frequently emerged during adulthood (30%), adolescence (19%), or late childhood (20%), with 20% of patients lacking information on systemic onset age. When both onset ages were available, dermatological manifestations preceded systemic features in 10% of patients, occurred in the same developmental category in 38%, and appeared later in 4%; in the remaining cases, onset was reported for only one manifestation type or not reported for either. Diagnostic delay was highly variable, with information available for 47 patients. Among these, moderate delays (6–10 years) were most common (13%), followed by long delays (7%) and very long delays (>20 years, 5%), whereas immediate diagnosis (≤1 year) occurred in only 1% of cases. Delay information was not reported in 68% of patients overall.

**Table 3:** Detailed patient profile characteristics.

Characteristic	Total (*N* = 146)
	*N* (%)
Sex	
Male	100 (68)
Female	46 (32)
Dermatological manifestation onset age	
Infancy (0–2 years)	1 (1)
Early childhood (3–6 years)	7 (5)
Late childhood (7–12 years)	22 (15)
Adolescence (13–17 years)	36 (25)
Young adulthood (18–29 years)	9 (6)
Adulthood (30–49 years)	11 (8)
Late adulthood (≥50 years)	0
Not reported	60 (41)
Systemic manifestation onset age	
Infancy (0–2 years)	0
Early childhood (3–6 years)	7 (5)
Late childhood (7–12 years)	29 (20)
Adolescence (13–17 years)	28 (19)
Young adulthood (18–29 years)	8 (5)
Adulthood (30–49 years)	44 (30)
Late adulthood (≥50 years)	1 (1)
Not reported	29 (20)
Relative timing of dermatological vs. systemic onset	
Dermatological onset earlier	15 (10)
Same onset category	56 (38)
Dermatological onset later	6 (4)
Only dermatological onset reported	9 (6)
Only systemic onset reported	40 (27)
Onset not reported for either	20 (14)
Diagnostic delay	
Immediate (≤1 year)	2 (1)
Short delay (2–5 years)	9 (6)
Moderate delay (6–10 years)	19 (13)
Long delay (11–20 years)	10 (7)
Very long delay (>20 years)	7 (5)
Not reported	99 (68)

Percentages were calculated using the 146 individual patients extracted from case reports and case series as the denominator. Percentages may not sum to 100% due to rounding.

Among the 44 patients with typical dermatological manifestations for whom diagnostic delay was reported, moderate delays (6–10 years) were the most frequently observed category (41%) (Table 4). This was followed by short delays (2–5 years, 20%), long delays (11–20 years, 18%), and very long delays (>20 years, 16%). Immediate diagnosis (≤1 year) was documented in only 5% of cases. When diagnostic delay was described according to sex, moderate delays (6–10 years) were the most frequently reported category among males (49%), whereas no female cases were reported in this category. Among females, very long delays (>20 years) represented the largest proportion of reported cases (43%). These descriptive findings should be interpreted with caution given the small number of female patients and the absence of formal statistical comparisons.

**Table 4:** Diagnostic delay among patients with typical dermatological manifestations, stratified by sex and restricted to cases with reported delay.

Diagnostic delay category	Total (*N* = 44)	Male (*N* = 37)	Female (*N* = 7)
	*N* (%)	*N* (%)	*N* (%)
Immediate (≤1 year)	2 (5)	1 (3)	1 (14)
Short delay (2–5 years)	9 (20)	7 (19)	2 (29)
Moderate delay (6–10 years)	18 (41)	18 (49)	0 (0)
Long delay (11–20 years)	8 (18)	7 (19)	1 (14)
Very long delay (>20 years)	7 (16)	4 (11)	3 (43)

The table includes only patients with typical dermatological manifestations and available diagnostic delay information (*n* = 44). Three additional patients with available diagnostic delay were excluded because they did not present typical dermatological manifestations. Percentages may not sum to 100% due to rounding.

When diagnostic delay was described according to the presence or absence of angiokeratomas among patients with available diagnostic delay information (Table 5), immediate diagnosis was reported only among patients with angiokeratomas (6%). Short delays were observed in 21% of patients without angiokeratomas and 18% of those with angiokeratomas, whereas moderate delays accounted for 43% and 39% of cases, respectively. Long delays (11–20 years) were reported in 21% of patients in both groups, while very long delays (>20 years) were observed in 14% of patients without angiokeratomas and 15% of those with angiokeratomas. When diagnostic delay was described according to hypohidrosis status (Table 5), immediate diagnosis was reported only among patients without hypohidrosis (7%), whereas no cases of immediate diagnosis were reported among those with hypohidrosis. Moderate delays (6–10 years) accounted for 65% of cases among patients with hypohidrosis and 27% among those without hypohidrosis. Long delays (11–20 years) were reported in 27% of patients without hypohidrosis and 12% of those with hypohidrosis, while very long delays (>20 years) accounted for 20% and 6% of cases, respectively. As this review was based exclusively on descriptive analyses, no formal statistical comparisons between groups were performed.

**Table 5:** Diagnostic delay according to specific dermatological manifestations (angiokeratomas and hypohidrosis), restricted to cases with reported delay.

Diagnostic delay category	Absent, *N* (%)	Present, *N* (%)
Angiokeratomas		
Immediate (≤1 year)	0 (0)	2 (6)
Short delay (2–5 years)	3 (21)	6 (18)
Moderate delay (6–10 years)	6 (43)	13 (39)
Long delay (11–20 years)	3 (21)	7 (21)
Very long delay (>20 years)	2 (14)	5 (15)
**Total (*N*)**	**14**	**33**
Hypohidrosis		
Immediate (≤1 year)	2 (7)	0 (0)
Short delay (2–5 years)	6 (20)	3 (18)
Moderate delay (6–10 years)	8 (27)	11 (65)
Long delay (11–20 years)	8 (27)	2 (12)
Very long delay (>20 years)	6 (20)	1 (6)
**Total (*N*)**	**30**	**17**

Percentages were calculated within each dermatological manifestation category using patients with available diagnostic delay information as the denominator. Percentages may not sum to 100% due to rounding.

## 4 Discussion

This scoping review aims to delineate the role of dermatological manifestations in the early diagnosis of FD. The clinical relevance of this topic is evident for patients in whom systemic involvement is significant and progressive, and diagnostic delay leads to disease progression that is extremely detrimental to patients (41). Furthermore, as infusion therapies are available that either replace the deficient enzyme or reduce its misfolding, a timely diagnosis may enable earlier treatment initiation, with the potential to slow disease progression and limit the development of irreversible organ damage (42).

Epidemiological studies, including data from the Fabry Outcome Survey (FOS), have demonstrated an association between cutaneous lesions and disease severity, suggesting that early dermatological recognition could facilitate timely diagnosis (36).

In the present review, the overall analysis included reported data from 2,209 patients, with patient-level characteristics extracted from 146 individual patients. Of note, a dermatologist was involved in the diagnostic process of FD in 66% of studies and 69% reported a potential contribution of dermatological assessment to earlier disease recognition. These findings should be interpreted as reflecting the characteristics of the published literature rather than routine clinical practice. Because this review specifically included studies focusing on dermatological manifestations of FD, the reported involvement of dermatologists is likely influenced by the review eligibility criteria and by publication bias, particularly considering the predominance of case reports and case series. Angiokeratomas and hypohidrosis were the most frequently reported cutaneous features, described in 88% and 50% of records, respectively. These features, although present in numerous other pathological conditions, may guide the diagnosis when occurring within a clinical history typical of FD (43, 44).

Previous reviews have described the spectrum and prevalence of cutaneous manifestations in FD. However, the potential role of these dermatological features in facilitating earlier recognition and their relationship with diagnostic timing has been only partially explored (45). By specifically examining the relationship between dermatological manifestations and diagnostic timing, the present review provides additional insights into the clinical relevance of cutaneous findings within the diagnostic pathway of FD.

Skin biopsy or histopathological examination was the most frequently reported dermatological investigative technique, being described in 59% of the included studies. This finding reflects the diagnostic approaches reported in the published literature and should not be interpreted as indicating that skin biopsy represents the principal diagnostic method for FD. Interestingly, in this cohort no differences in diagnostic delay were observed according to the presence or absence of angiokeratomas (diagnostic delay ≤1–10 years: 63% vs. 64%, respectively). In contrast, among patients with available diagnostic delay information, a higher proportion of those with hypohidrosis had a reported diagnostic delay of ≤10 years compared with those without hypohidrosis (83% vs. 54%, respectively). These descriptive patterns should be considered in the context of the diagnostic pathway, in which dermatologists may not always be involved at an early stage and, consequently, may have limited opportunities to contribute to earlier disease recognition (29, 44, 46, 47). Moreover, as this review includes exclusively patients with dermatological manifestations, descriptive comparisons across different types of cutaneous involvement cannot establish whether these manifestations are associated with earlier diagnosis. On the other hand, an indirect contextual comparison with a global observational registry suggests that the diagnostic delays reported among patients with dermatological manifestations in the present review appear shorter than those observed in the registry. However, this comparison should be interpreted with caution because of the substantial differences between the study populations and the absence of an appropriate comparator group (48). In this registry, symptom onset occurred at 9 and 13 years of age in affected males and females, respectively, whereas FD was diagnosed at 23 and 32 years, respectively, corresponding to a diagnostic delay of 14 years in males and 19 years in females (48). Notably, in the case series included in this review, 66% of patients presenting with dermatological manifestations had a reported diagnostic delay ranging from 1 to 10 years (immediate: ≤1 year; moderate: 6–10 years). Although these descriptive findings suggest that dermatological manifestations may contribute to earlier recognition of FD, they should be interpreted with caution given the absence of an appropriate comparator group and the methodological limitations of this review.

As expected for an X-linked disorder, the disease predominantly affects males (68%); however, females may also manifest the disease due to the lyonization phenomenon, with highly variable severity and in most cases subtle symptoms (49). In this context, among males, who typically present with more severe disease, 71% of patients with dermatological manifestations experienced a diagnostic delay of 1 to 10 years. In contrast, only 43% of females exhibited a diagnostic delay within the same time range.

Thus, dermatological assessment may represent a relevant component of the diagnostic pathway, particularly considering that dermatologic manifestations in more than half of the reported cases precede (10%), are exclusive (6%), or occur concomitantly (38%) with systemic manifestations. Moreover, the frequent reporting of dermatologic manifestations in younger patients (46%; infancy, early childhood, late childhood, and adolescence) suggests that dermatologists may have an opportunity to recognize suggestive features during the early phases of the disease. In this context, dermatological findings should not be regarded as isolated manifestations, but rather as potential early clues to an underlying multisystem disorder. A high index of suspicion among pediatric dermatologists may therefore facilitate timely referral for biochemical and genetic evaluation, enabling diagnosis and appropriate management at a stage when major organ involvement may still be preventable or attenuated. This represents a particularly important diagnostic window, as the progressive accumulation of Gb3 and related substrates can lead to irreversible renal and cardiac damage over time. These findings highlight the potential importance of integrating dermatological evaluation within structured diagnostic pathways and a multidisciplinary approach, which may facilitate earlier recognition and referral for confirmatory testing.

The estimated incidence of FD in the general population is 1 in 117,000 (50) and 1 in 40,000 males (51). However, FD is probably underdiagnosed, as illustrated by newborn screening analyses that provide overall incidence of *GLA* variants in approximately 1/1,250–3,100 males (52, 53). This discrepancy between clinically diagnosed cases and screening-based estimates further supports the hypothesis that FD remains substantially underrecognized in clinical practice. In this context, careful evaluation of suggestive dermatological findings may contribute to earlier recognition of the disease. In particular, among systemic manifestations, gastrointestinal (91%) and neurological (88%) symptoms were the most frequently reported, suggesting that they should be actively investigated alongside dermatologic findings during clinical history taking. Beyond these specific symptom combinations, a thorough personal and family history is essential. Dermatologists should systematically inquire about recurrent or unexplained symptoms commonly associated with FD in both the patient and their family members, as a multisystemic pattern or a suggestive family history may provide an important clue to the diagnosis.

This review has several limitations that should be acknowledged. First, according to the PCC framework, the inclusion criteria were specifically designed to identify studies reporting dermatological manifestations in patients with FD. Consequently, the review does not include a comparator population without cutaneous involvement, introducing an inherent selection bias. This methodological choice limits the generalizability of the findings and should be taken into account when interpreting the potential contribution of dermatological manifestations to earlier diagnosis. Second, only studies published in English were included, which may have introduced language bias and resulted in the exclusion of relevant evidence published in other languages, particularly from countries with extensive experience in FD research. Third, the included studies showed substantial heterogeneity in study designs, patient populations, outcome definitions, and reporting methods, limiting the comparability of findings across studies and supporting a descriptive interpretation of the available evidence. In particular, study-level frequencies reflect the distribution of findings across heterogeneous publications rather than pooled epidemiological estimates and should therefore not be interpreted as estimates of the prevalence of dermatological manifestations or their diagnostic contribution. In addition, patient-level data were available only for a subset of the included studies. Finally, publication bias cannot be excluded, as approximately three-quarters of the included studies were case reports or case series, study designs that are inherently more likely to describe unusual, clinically evident, or diagnostically informative presentations. Therefore, the findings of this review should be interpreted with appropriate caution, taking into account the potential influence of publication bias.

Overall, the available evidence suggests that recognition of suggestive dermatological manifestations may facilitate earlier suspicion of FD and timely referral for confirmatory evaluation. Integrating dermatological assessment within multidisciplinary diagnostic pathways may therefore contribute to earlier disease recognition, particularly when cutaneous findings occur alongside other compatible systemic manifestations. However, the extent to which dermatological assessment influences diagnostic delay or subsequent clinical outcomes cannot be established from the predominantly descriptive evidence included in this review and should be evaluated in future comparative studies.
