# Supplementary material for: The role of dermatological manifestations in the early diagnosis of Fabry disease: a scoping review
Source: Front Med (Lausanne). 2026 Oct 6;13:1899166. doi: 10.3389/fmed.2026.1899166 (PMC13683945; doi:10.3389/fmed.2026.1899166)
Supplement: Supplementary file 2 [file Table2.docx]

**Supplementary Table 2.** Patient-level characteristics extracted from case reports and case series.

| **References** | **Authors, Year** | **Patient ID** | **Sex** | **Dermatological manifestations** | **Onset age dermatological manifestation** | **Other systems involved** | **Onset age other systems involved** | **Dermatology diagnostic methods** | **Diagnostic delay** |
| --- | --- | --- | --- | --- | --- | --- | --- | --- | --- |
| [9] | Ahlmén et al., 1982 | 1 | M | • Hypohidrosis • Skin lesion | NA | • Neurological • Renal | Young adulthood | NA | Short delay |
|  |  | 2 | M | • Hypohidrosis | Late childhood | • Neurological • Renal • Cardiovascular • Ocular | Late childhood | NA | Moderate delay |
|  |  | 3 | M | • Hypohidrosis • Papules | Adolescence | • Neurological • Renal • GI | Late childhood | NA | Moderate delay |
| [11] | Choudhury et al., 2005 | 1 | M | • Angiokeratomas (atypical widespread distribution) | Early childhood | • Neurological | Late childhood | • Clinical examination • Biopsy | Moderate delay |
| [12] | Corry et al., 2011 | 1 | M | • Angiokeratoma (solitary) | NA | • Renal | NA | • Clinical examination • Biopsy | NA |
| [13] | Da Silva et al., 2014 | 1 | M | • Angiokeratomas | Early childhood | • Neurological • Renal • Ocular • GI • Auditory | Childhood | • Biopsy | Moderate delay |
|  |  | 2 | M | • Angiokeratomas | Childhood | • Neurological • Renal • Ocular | Childhood | • Clinical examination | Long delay |
|  |  | 3 | F | NA | NA | • Neurological • Renal • Ocular • Auditory | Adulthood | NA | NA |
| [14] | de Groot, 1970 | 1 | M | • Angiokeratomas | Late childhood | • Neurological • Ocular | Late childhood | NA | Long delay |
|  |  | 2 | M | NA | NA | • Neurological • Ocular | Late childhood | NA | NA |
|  |  | 3 | M | • Angiokeratomas | Late childhood | • Neurological • Ocular | Late childhood | NA | Immediate diagnosis |
|  |  | 4 | M | • Angiokeratomas | Adolescence | • Neurological • Ocular | Childhood | • Biopsy | Short delay |
|  |  | 5 | M | • Angiokeratomas | Adolescence | • Ocular | NA | NA | Moderate delay |
|  |  | 6 | F | • Angiokeratomas | Late childhood | • Neurological • Ocular | Late childhood | NA | Immediate diagnosis |
|  |  | 7 | F | NA | NA | • Ocular | NA | NA | NA |
|  |  | 8 | F | NA | NA | • Ocular | NA | NA | NA |
|  |  | 9 | F | • Angiokeratomas | Young adulthood | • Ocular | NA | NA | NA |
|  |  | 10 | F | • Angiokeratomas | Young adulthood | • Neurological • Ocular | Young adulthood | NA | Short delay |
|  |  | 11 | F | • Angiokeratomas | Young adulthood | • Ocular | NA | NA | NA |
|  |  | 12 | F | • Angiokeratomas | Adolescence | • Ocular | NA | NA | NA |
| [15] | de Jesus et al., 2018 | 1 | F | • Genital angiokeratoma | Late adulthood | • Neurological | Childhood | • Dermoscopy • Biopsy | NA |
| [16] | Dean & Starr, 2014 | 1 | M | • Lymphedema | Adulthood | • Neurological • Renal • GI • Ocular • Cardiovascular | Adulthood | NA | Long delay |
| [17] | Dilorenzo et al., 1969 | 1 | M | • Angiokeratomas (bathing-trunk) | Late childhood | • Cerebrovascular • Cardiac • Neurological • Ocular • Renal | Adulthood | • Biopsy | NA |
| [18] | Franques et al., 2017 | 1 | M | NA | NA | • Cardiac | Adulthood | NA | NA |
|  |  | 2 | M | NA | NA | • Cardiac | Adulthood | NA | NA |
|  |  | 3 | M | NA | NA | • Cardiac • Ocular | Adulthood | NA | NA |
|  |  | 4 | M | • Hypohidrosis | NA | • Renal • Cardiac | Adulthood | NA | NA |
|  |  | 5 | M | • Hypohidrosis | NA | • Renal | Adulthood | NA | NA |
|  |  | 6 | M | • Hypohidrosis | NA | • Cardiac | Adulthood | NA | NA |
|  |  | 7 | M | • Hypohidrosis • Angiokeratomas | NA | • Renal • Cardiac | Adulthood | NA | NA |
|  |  | 8 | M | • Hypohidrosis • Angiokeratomas | NA | • Cardiac | Adulthood | NA | NA |
|  |  | 9 | M | • Hypohidrosis | NA | NA | Adulthood | NA | NA |
|  |  | 10 | M | • Hypohidrosis | NA | • Cardiac | Adulthood | NA | NA |
|  |  | 11 | F | NA | NA | • Cardiac • Ocular | Adulthood | NA | NA |
|  |  | 12 | M | • Hypohidrosis | NA | • Cardiac • Ocular | Adulthood | NA | NA |
|  |  | 13 | M | • Hypohidrosis | NA | • Ocular | Adulthood | NA | NA |
|  |  | 14 | M | • Hypohidrosis | NA | • Cardiac • Ocular | Adulthood | NA | NA |
|  |  | 15 | M | NA | NA | • Cardiac | Adulthood | NA | NA |
|  |  | 16 | M | • Hypohidrosis • Angiokeratomas | NA | • Cardiac • Ocular | Adulthood | NA | NA |
|  |  | 17 | F | • Hypohidrosis | NA | • Ocular • GI | Adulthood | NA | NA |
|  |  | 18 | F | • Hypohidrosis | NA | • Ocular | Adulthood | NA | NA |
| [19] | Furujo et al., 2013 | 1 | M | • Angiokeratomas • Hypohidrosis | Childhood | • Neurological | Childhood | • Clinical examination | Moderate delay |
|  |  | 2 | M | • Hypohidrosis/anhidrosis | Childhood | • Neurological • Ocular • GI | Childhood | • Clinical examination | NA |
| [20] | Galanos et al., 2002 | 1 | M | • Angiokeratomas | Adolescence | • Neurological • Renal | Adolescence | • Clinical examination • Biopsy | Short delay |
|  |  | 2 | M | • Angiokeratomas | Young adulthood | • Renal | Adulthood | • Clinical examination | NA |
|  |  | 3 | M | • Angiokeratomas | Late childhood / Adolescence | • Renal | Adulthood | • Clinical examination • Biopsy | Moderate delay |
|  |  | 4 | M | • Angiokeratomas | NA | • Neurological • Renal | Adulthood | • Clinical examination | NA |
|  |  | 5 | F | NA | NA | • Renal • Ocular | Adulthood | NA | NA |
|  |  | 6 | M | • Angiokeratomas | Adolescence | • Neurological • Renal | Adolescence | • Clinical examination | NA |
|  |  | 7 | M | • Angiokeratomas | Adolescence | • Neurological | Adolescence | • Clinical examination | NA |
|  |  | 8 | F | NA | NA | • Renal • Ocular | Adulthood | NA | NA |
|  |  | 9 | M | • Angiokeratomas | Adolescence | • Neurological • Renal | Adolescence | • Clinical examination | NA |
|  |  | 10 | M | • Angiokeratomas | Adolescence | • Neurological • Cardiac | Adolescence | • Clinical examination | NA |
|  |  | 11 | M | • Angiokeratomas | Adolescence | • Neurological • Renal | Adolescence | • Clinical examination | NA |
|  |  | 12 | M | • Angiokeratomas | Adolescence | • Renal • Cardiac | Adulthood | • Clinical examination | NA |
|  |  | 13 | M | • Angiokeratomas | Adolescence | • Neurological • Renal | Adolescence | • Clinical examination | NA |
|  |  | 14 | F | • Angiokeratomas | Adulthood | • Ocular | Adulthood | NA | NA |
|  |  | 15 | M | • Angiokeratomas | Adolescence | • Renal • Cardiac | Adulthood | • Clinical examination | NA |
|  |  | 16 | M | • Angiokeratomas | Adolescence | • Neurological • Renal | Adolescence | • Clinical examination | NA |
|  |  | 17 | M | • Angiokeratomas | Adolescence | • Neurological • Renal | Adolescence | • Clinical examination | NA |
|  |  | 18 | M | • Angiokeratomas | Adolescence | • Neurological • Renal | Adolescence | • Clinical examination | NA |
| [21] | Hsu et al., 2019 | 1 | M | • Hypohidrosis • Angiokeratomas (genital) • Telangiectasia | Adolescence | • Neurological • GI • Autonomic • Ocular • Cerebrovascular | Childhood | • Biopsy | Moderate delay |
| [22] | Jung et al., 2015 | 1 | M | • Hypohidrosis • Angiokeratomas | Infancy | • Neurological | Adolescence | • Biopsy | Moderate delay |
| [23] | Kotnik et al., 2005 | 1 | M | • Angiokeratomas (diffuse) • Hypohidrosis | Adolescence | • Neurological • Musculoskeletal • Cardiac • Renal • Ocular • Cerebrovascular | Young adulthood | • Biopsy | Very long delay |
| [24] | Larralde et al., 2004 | 1 | M | • Angiokeratomas • Hypohidrosis | Adolescence | • Neurological • Ocular • GI | Early childhood | • Biopsy • EM | Moderate delay |
|  |  | 2 | M | • Hypohidrosis | NA | • Neurological • Ocular • GI | Early childhood | • EM | Moderate delay |
|  |  | 3 | M | • Angiokeratomas • Hypohidrosis | Adolescence | • Neurological • Ocular • GI | Late childhood | • EM | Moderate delay |
|  |  | 4 | M | • Angiokeratomas • Hypohidrosis | Adolescence | • Neurological • Ocular • GI | Early childhood | • EM | Long delay |
|  |  | 5 | M | • Angiokeratomas • Hypohidrosis | NA | • Neurological • Ocular • GI | Early childhood | • EM | Long delay |
|  |  | 6 | M | • Angiokeratomas • Hypohidrosis | NA | • Neurological • Renal • Ocular | Childhood | • EM | NA |
|  |  | 7 | F | • Angiokeratomas | Adulthood | • Ocular | Adulthood | • EM | NA |
|  |  | 8 | F | • Angiokeratomas | Adulthood | • Ocular | Adulthood | • EM | NA |
|  |  | 9 | F | NA | NA | • Ocular | Adulthood | • EM | NA |
|  |  | 10 | F | NA | NA | • Ocular | Adulthood | • EM | NA |
|  |  | 11 | F | NA | NA | NA | NA | • EM | NA |
| [25] | Li et al., 2023 | 1 | M | • Angiokeratomas • Anhidrosis | Childhood | • Ocular • GI • Auditory • Endocrine | Childhood | • Clinical examination | Moderate delay |
|  |  | 2 | M | • Hypohidrosis | Childhood | • Renal • GI | Childhood | • Clinical examination | Moderate delay |
|  |  | 3 | M | • Angiokeratomas | Childhood | • Renal • Cardiac • GI | Childhood | • Clinical examination | Short delay |
|  |  | 4 | M | • Hypohidrosis | Late childhood | • Neurological • Renal • GI | Late childhood | • Clinical examination | Short delay |
|  |  | 5 | M | • Angiokeratomas • Hypohidrosis | Childhood | • Neurological • Ocular • GI • Musculoskeletal • Endocrine | Childhood | • Clinical examination | Moderate delay |
|  |  | 6 | M | • Angiokeratomas • Anhidrosis | Childhood | • Ocular • GI | Childhood | • Clinical examination | Moderate delay |
|  |  | 7 | F | • Angiokeratomas | Childhood | • GI • Endocrine | Childhood | • Clinical examination | Short delay |
| [26] | Lidove et al., 2006 | 1 | F | • Hyperhidrosis | Early childhood | • Gastrointestinal • Ocular • Neurological • Cardiac | Adulthood | • Clinical examination | Very long delay |
|  |  | 2 | F | • Hyperhidrosis | Early childhood | • Ocular | Adolescence | • Clinical examination | Very long delay |
|  |  | 3 | M | • Hyperhidrosis | Late childhood | • Gastrointestinal • Ocular • Neurological • Auditory | Adolescence | • Skin biopsy | Moderate delay |
|  |  | 4 | M | • Hyperhidrosis | Adulthood | • Cardiac • Ocular • Musculoskeletal | Adulthood | • Clinical examination | Short delay |
|  |  | 5 | F | • Hyperhidrosis • Angiokeratomas | Early childhood | • Cardiac • Ocular | Adulthood | • Clinical examination | Long delay |
| [27] | Lidove et al., 2012 | 1 | M | • Angiokeratomas • Raynaud’s phenomenon | Adulthood | • Ocular • Neurological • Auditory • Cardiac | Adulthood | • Clinical examination | Long delay |
|  |  | 2 | F | • Angiokeratomas | Adulthood | • Ocular • Neurological • Auditory • Renal • Cardiac | Adulthood | • Clinical examination | Very long delay |
|  |  | 3 | M | • Angiokeratomas | Young adulthood | • Neurological • Ocular | Adolescence | • Clinical examination | Very long delay |
|  |  | 4 | M | NA | NA | • Renal • Auditory | Adulthood | NA | Moderate delay |
| [28] | Lorenz et al., 2003 | 1 | M | • Angiokeratomas | NA | • Neurological • Renal • Cardiac • Ocular | Childhood | NA | NA |
|  |  | 2 | M | • Angiokeratomas | NA | • Neurological • Renal • Cardiac • Ocular | Adulthood | NA | NA |
|  |  | 3 | M | • Angiokeratomas | NA | • Neurological • Renal • Cardiac • Ocular | NA | NA | NA |
|  |  | 4 | M | • Angiokeratomas | NA | • Neurological • Renal • Ocular • Auditory | Early childhood | NA | NA |
|  |  | 5 | M | NA | NA | • Renal • Cardiac • Ocular | Adulthood | NA | NA |
|  |  | 6 | M | • Angiokeratomas | Childhood | • Neurological • Renal • Cardiac • Ocular • Auditory | Late childhood | NA | NA |
|  |  | 7 | F | • Angiokeratomas | NA | • Neurological • Renal • Cardiac • Ocular | NA | NA | NA |
|  |  | 8 | F | NA | NA | • Cardiac • Ocular | Adulthood | NA | NA |
| [29] | Marchesoni et al., 2010 | 1 | M | • Angiokeratomas (initially misdiagnosed as petechiae) | Late childhood | • Neurological • Gastrointestinal | Late childhood | • Clinical examination | Long delay |
|  |  | 2 | M | • Angiokeratomas | Early childhood | • Neurological | Late childhood | • Clinical examination | Very long delay |
|  |  | 3 | M | • Skin findings not further specified | NA | • Neurological • Renal | Late childhood | NA | Long delay |
| [30] | Martin et al., 2007 | 1 | F | • Digital ischemia with non-blanching purple discoloration | NA | • Neurological • Cardiovascular • GI • Family history | NA | • Biopsy • EM | NA |
| [32] | Menkes, 1999 | 1 | M | • Angiokeratoma corporis diffusum | Late childhood | • Neurological  • Renal • Ocular • Autonomic | Late childhood/Adulthood | • Biopsy • EM | NA |
| [33] | Mungan et al., 2015 | 1 | M | • Angiokeratomas • Hypohidrosis/anhidrosis | Late childhood | • Musculoskeletal • Autonomic | Late childhood | • Clinical examination • Biopsy | ~2 years |
| [34] | Nagai-Sangawa et al., 2022 | 1 | M | • Hypohidrosis • Angiokeratomas | Late childhood | • Neurological • Renal • Ocular | Late childhood | • Clinical examination • Biopsy | Moderate delay |
|  |  | 2 | M | • Hypohidrosis • Angiokeratomas | Late childhood | • Neurological • Ocular | Late childhood | • Clinical examination | NA |
|  |  | 3 | M | • Hypohidrosis • Angiokeratomas | Late childhood | • Neurological • Renal • Ocular | Adolescence | • Clinical examination | NA |
|  |  | 4 | M | • Hypohidrosis • Angiokeratomas | Late childhood | • Neurological • Ocular | Late childhood | • Clinical examination | NA |
|  |  | 5 | F | • Hypohidrosis • Angiokeratomas | Adulthood | • Cardiac | Adulthood | • Clinical examination | NA |
| [35] | Nelis & Jacobs, 1989 | 1 | M | • Angiokeratomas | Adulthood | • GI • Neurological • Ocular | Young adulthood | Clinical examination | Very long delay |
|  |  | 2 | M | • Angiokeratomas | Adulthood | • Neurological • Dental | Adulthood | Clinical examination | NA |
| [37] | Ries et al., 2003 | 1 | M | NA | NA | • Gastrointestinal | Early childhood | • Clinical examination | NA |
|  |  | 2 | M | • Hypohidrosis | Early childhood | • Neurological | Early childhood | • Clinical examination | NA |
|  |  | 3 | M | • Angiokeratomas • Hypohidrosis | Late childhood | • Neurological • Ocular | Late childhood | • Clinical examination | NA |
|  |  | 4 | M | • Hypohidrosis | Late childhood | • Gastrointestinal • Ocular | Late childhood | • Clinical examination | NA |
|  |  | 5 | M | • Hypohidrosis | Adolescence | • Neurological • Ocular | Adolescence | • Clinical examination | NA |
|  |  | 6 | M | • Angiokeratomas • Hypohidrosis | Adolescence | • Neurological • Gastrointestinal • Ocular | Adolescence | • Clinical examination | NA |
|  |  | 7 | M | • Hypohidrosis | Adolescence | • Neurological • Ocular | Adolescence | • Clinical examination | NA |
|  |  | 8 | M | • Angiokeratomas • Hypohidrosis | Adolescence | • Neurological • Ocular | Adolescence | • Clinical examination | NA |
|  |  | 9 | M | • Angiokeratomas • Hypohidrosis | Adolescence | • Neurological • Ocular | Adolescence | • Clinical examination | NA |
|  |  | 10 | M | • Angiokeratomas • Hypohidrosis | Adolescence | • Neurological • Renal • Ocular | Adolescence | • Clinical examination | NA |
|  |  | 11 | M | • Angiokeratomas • Hypohidrosis | Adolescence | • Neurological | Adolescence | • Clinical examination | NA |
|  |  | 12 | M | • Hypohidrosis | Young adulthood | • Neurological • Ocular | Young adulthood | • Clinical examination | NA |
|  |  | 13 | M | • Angiokeratomas • Hypohidrosis | Young adulthood | • Neurological • Gastrointestinal • Cardiac • Renal | Young adulthood | • Clinical examination | NA |
|  |  | 14 | M | • Angiokeratomas • Hypohidrosis | Young adulthood | • Neurological • Gastrointestinal • Cardiac • Ocular • Renal | Young adulthood | • Clinical examination | NA |
|  |  | 15 | M | • Angiokeratomas • Hypohidrosis | Young adulthood | • Neurological • Gastrointestinal • Cardiac • Renal • Ocular | Young adulthood | • Clinical examination | NA |
|  |  | 16 | F | • Angiokeratomas • Hypohidrosis | Late childhood | • Neurological • Ocular | Late childhood | • Clinical examination | NA |
|  |  | 17 | F | NA | NA | • Ocular | Late childhood | • Clinical examination | NA |
|  |  | 18 | F | NA | NA | NA | NA | NA | NA |
|  |  | 19 | F | • Angiokeratomas | Late childhood | • Neurological • Ocular | Late childhood | • Clinical examination | NA |
|  |  | 20 | F | NA | NA | • Neurological • Ocular | Late childhood | • Clinical examination | NA |
|  |  | 21 | F | NA | NA | • Renal • Ocular | Late childhood | • Clinical examination | NA |
|  |  | 22 | F | • Hypohidrosis | Late childhood | • Neurological • Ocular | Late childhood | • Clinical examination | NA |
|  |  | 23 | F | • Angiokeratomas | Late childhood | • Renal • Ocular | Late childhood | • Clinical examination | NA |
|  |  | 24 | F | • Angiokeratomas | Late childhood | • Renal • Ocular | Late childhood | • Clinical examination | NA |
|  |  | 25 | F | • Angiokeratomas | Adolescence | • Gastrointestinal • Renal • Ocular | Adolescence | • Clinical examination | NA |
|  |  | 26 | F | • Angiokeratomas | Adolescence | • Cardiac • Ocular | Adolescence | • Clinical examination | NA |
|  |  | 27 | F | • Angiokeratomas • Hypohidrosis | Adolescence | • Gastrointestinal • Renal • Ocular | Adolescence | • Clinical examination | NA |
|  |  | 28 | F | • Angiokeratomas • Hypohidrosis | Adolescence | • Neurological • Gastrointestinal • Cardiac • Renal • Ocular | Adolescence | • Clinical examination | NA |
|  |  | 29 | F | • Hypohidrosis | Adolescence | • Gastrointestinal • Renal | Adolescence | • Clinical examination | NA |
|  |  | 30 | F | • Angiokeratomas | Adolescence | • Cardiac • Ocular | Adolescence | • Clinical examination | NA |
|  |  | 31 | F | • Angiokeratomas | Adolescence | • Ocular | NA | • Clinical examination | NA |
|  |  | 32 | F | • Hypohidrosis | Adolescence | NA | NA | • Clinical examination | NA |
| [39] | Tokuriki et al., 2013 | 1 | M | • Papules • Cherry angiomas | NA | • Neurological • Autonomic • Family history | Late childhood | • Biopsy • EM | Long delay |
| [40] | Werninghaus et al., 1995 | 1 | M | • Telangiectases • Angiokeratomas | NA | • Renal • Cardiovascular • Endocrine • Auditory • Neurological • Pulmonary | Childhood | • Biopsy • EM | NA |

**Abbreviations:** GI = gastrointestinal; EM = electron microscopy; NA = not available.

**Notes:**

- **Age of onset (dermatological / other symptoms):**

Infancy = 0–2 years

Early childhood = 3–6 years

Late childhood = 7–12 years

Adolescence = 13–17 years

Young adulthood = 18–29 years

Adulthood = 30–49 years

Late adulthood = ≥50 years

- **Diagnostic delay:**

Immediate diagnosis (≤1 year)

Short delay = 2–5 years

Moderate delay = 6–10 years

Long delay = 11–20 years

Very long delay = >20 years
